# Uncovering the role of IFNAR1 in experimental cerebral malaria

**DOI:** 10.1186/1475-2875-9-S2-P3

**Published:** 2010-10-20

**Authors:** Elizabeth Ball, Carlos Penha Gonçalves

**Affiliations:** 1 Disease Genetics Group, Instituto Gulbenkian de Ciência, Oeiras, Portugal

## 

Cerebral malaria (CM) is a severe complicated form of malaria, resulting in an overwhelming inflammatory response in the brain due to infection from *Plasmodium-falciparum*[1]. Only a fraction of malaria infected individuals develop CM and it has been shown that both genetic and non-genetic factors determine CM susceptibility [2]. Variants in Interferon alpha receptor-1 (IFNAR1) have been associated with protection from CM in humans [3] and in susceptibility to severe forms of malaria, such as CM. As in humans, susceptibility of mice to CM is determined by genetic factors (unpublished data, Sambo et al).

Type-I Interferon, α/β (IFN-I), plays a key role in regulating the immune response through release of pro- and anti-inflammatory cytokines and stimulating antigen presentation and cellular cytotoxicity. Its expression has been found to be up regulated upon infection with viruses and bacteria, and can illicit protective or disease-aggravating effects [4]. From this knowledge we hypothesize that IFNAR1 is a determinant of the inflammatory response that leads to development of Experimental cerebral malaria (ECM).

*Ifnar1-/-* mice were infected with *Plasmodium berghei* ANKA (*P.berghei* ANKA) and assessed for survival and development of ECM. 70% of mice did not develop ECM and died 28-30 days later from anemia and hyper-parasitemia. *Ifnar1-/-* mice do not show a decrease in parasite burden, and display mild blood brain barrier breakage. We are currently investigating brain vasculature and infiltration of inflammatory molecules within these mice and are conducting transfer experiments using *Ifnar1-/-* and *Rag2-/-*mice with the aim of defining the role of IFNAR1 in development of ECM upon infection with *P.berghei* ANKA, and determining where and how this protection is inflicted.
